# Validation and Improvement of a Convolutional Neural Network to Predict the Involved Pathology in a Head and Neck Surgery Cohort

**DOI:** 10.3390/ijerph191912200

**Published:** 2022-09-26

**Authors:** Dorian Culié, Renaud Schiappa, Sara Contu, Boris Scheller, Agathe Villarme, Olivier Dassonville, Gilles Poissonnet, Alexandre Bozec, Emmanuel Chamorey

**Affiliations:** 1 Head and Neck Surgery Department, Antoine Laccassagne Center, 06100 Nice, France; 2 Epidemiology, Biostatistics and Health Data Department, Antoine Laccassagne Center, 06100 Nice, France

**Keywords:** neural network, cohort constitution, patient classification

## Abstract

The selection of patients for the constitution of a cohort is a major issue for clinical research (prospective studies and retrospective studies in real life). Our objective was to validate in real life conditions the use of a Deep Learning process based on a neural network, for the classification of patients according to the pathology involved in a head and neck surgery department. 24,434 Electronic Health Records (EHR) from the first visit between 2000 and 2020 were extracted. More than 6000 EHR were manually classified in ten groups of interest according to the reason for consultation with a clinical relevance. A convolutional neural network (TensorFlow, previously reported by Hsu et al.) was then used to predict the group of patients based on their pathology, using two levels of classification based on clinically relevant criteria. On the first and second level of classification, macro-average performances were: 0.95, 0.83, 0.85, 0.97, 0.84 and 0.93, 0.76, 0.83, 0.96, 0.79 for accuracy, recall, precision, specificity and F1-score versus accuracy, recall and precision of 0.580, 580 and 0.582 for Hsu et al., respectively. We validated this model to predict the pathology involved and to constitute clinically relevant cohorts in a tertiary hospital. This model did not require a preprocessing stage, was used in French and showed equivalent or better performances than other already published techniques.

## 1. Introduction

Artificial intelligence (AI) is currently a major area of interest and research, particularly in the medical field and in oncology. Some teams have developed tools to assist in the diagnosis, choice of treatment and prognosis [1,2]. However, the establishment of cohorts of patient with a clinical interest, to build solid databases, is a mandatory prerequisite for any AI analysis.

Building patient cohorts around defined pathologies or phenotypes is a major challenge [3] in real word medical activity. Those cohorts, in addition to serving as input for AI algorithms, are also very useful to improve clinical trial recruitment [4,5], outcome prediction, survival analysis and carrying out real life and retrospective studies. They can also provide a reflection of the activity of a medical team [6,7]. The identification of patients from the beginning of their care for the construction of homogeneous cohorts is therefore a major issue, a request from medical teams and a lack in current clinical practice.

Different techniques for identifying patients of interest have been described, but a simple and rapid tool is urgently needed [8]. Other approaches have been developed to predict patient phenotypes by ICD codes (International Statistical Classification of Disease and Related Health Problems). Ferrao et al. [9] developed a data mining approach to support clinical coding in the ICD-9. In their study, they compared different approaches to predict ICD-9 codes. Venkataraman et al. [10] also compared different approaches to determine ICD-9 codes automatically in a cohort of human and veterinary records. They trained long short-term memory (LSTM) recurrent neural networks (RNNs) and compared them with decision tree and random forest techniques [11]. Tam et al. developed a high-quality procedure based on combined processes and on structured and unstructured data to create clinically defined EHR-derived cohorts. This process was initially built to identify patients with acute coronary syndrome (ACS) in patients in a large general hospital [12]. A review of the literature [13] summarizes all published approaches to automatically identify patients based on their phenotype. It lists different possibilities to perform automatic classification: natural language processing (NLP)-based techniques, rule-based systems, statistical analyses, data mining, machine learning and combined techniques. It thus appears that rule-based systems are gradually being abandoned in favor of AI approaches. In this review, no single AI technique was superior, and all had similar efficiencies. All also had their own weaknesses. Furthermore, none of these previously published techniques has been validated in the literature, such publications are needed before considering the use of these tools in routine clinical practice.

To our knowledge, none of these reported studies has been validated, either for the choice of technique, external reproducibility by other teams, on a different medical theme or in a different language. Convolutional Neural Networks have already been used to classify different aspects in medical fields, but few reports are available for patient identification based on the first medical report.

Head and neck oncology surgery involves the management of many different pathologies, and therefore of different patients. The grouping of these patients according to these subgroups is therefore an essential step before initiating any research project.

Our aim was to describe, evaluate and valid a rapid and useful automated process for identifying and classifying patients into clinically relevant groups, based on their reason for first consultation, in a head and neck oncology surgery department.

## 2. Materials and Methods

An Electronic Health Records (EHR) is a multimedia file that can be electronically stored, transmitted and managed. EHR of a medical visit is a text dictated by the surgeon to transcribe the interview. It includes the reason for the visit, the patient’s complaints, the examinations carried out and the clinical examination of the patient. The data used in this study were text files of the EHR of the first visit. All files were free text in a French language. The files were previously pseudonymized for the patient and surgeon names.

Head and neck oncology surgery in our institution covers different fields. It includes thyroid and parathyroid gland surgery, skin cancer surgery, salivary gland surgery, upper aero-digestive tract (UADT) cancer management. UADT cancers include oral cavity, hypopharyngeal, laryngeal, oropharyngeal, nasopharyngeal and nasal cavity cancers (and all cervical masses).

EHR of all patients who had a consultation with one of the surgeons of the head and neck surgery department between 2000 and 2020 were extracted.

EHR of the first visit were then selected. A subset of the selected EHR was randomly chosen and manually categorized by expert (head and neck surgeons) into 10 clinically relevant group based on the reason for consultation:

- “Thyroid and parathyroid pathology”: thyroid nodules, thyroid pathology (*Basedow* and *Hashimoto’s* disease), parathyroid dysfunction;
- “Salivary gland pathology”: major salivary gland or accessory salivary gland tumor;
- “Head and neck skin pathology”: squamous cell carcinoma, basocellular carcinoma or melanoma located in the head and neck area;
- “Oral cavity”: oral cavity cancer;
- “Hypopharynx/larynx”: hypopharyngeal cancer, laryngeal cancer;
- “Oropharynx”: oropharyngeal cancer;
- “Nasopharynx”: nasopharyngeal cancer;
- “Isolated cervical lymph nodes”: cervical nodes with unknown primary;
- “Nasal cavity and sinuses”: nasal cavity and paranasal sinuses;
- “Other”: all the other reasons for the consultation.

Those EHR with manually labelled group constituted the initial dataset for this study.

We did not perform any preprocessing step. The entire project was done in Python. We used the similar deep learning model described by Hsu et al. [14]. Briefly, a deep neural network was built using the Keras Python deep learning library running on top of the TennsorFlow deep learning framework. All hyperparameters and codes are available in the publication by Hsu et al. [14]. In short, the convolutional layer is preceded by a word embedding and tokenizer layer. The filter size was set to 5 × 400, and the stride was set to 1. As it is compulsory to define a priori a modality of activation of each neuron, we chose the commonly used ReLu (Rectified Linear Unit). When training our model, we used Adam Optimizer, a parameter of the Keras algorithm that is an alternative optimization algorithm that provides more efficient neural network weights by running repeated cycles of adaptative moment estimation, with a moderate learning rate to avoid overfitting. An epoch of 30 was used to give the model sufficient time to complete convergence.

The dataset was randomly divided in a training set of 80% of the dataset and the remaining 20% for the test set.

**Figure 1:**
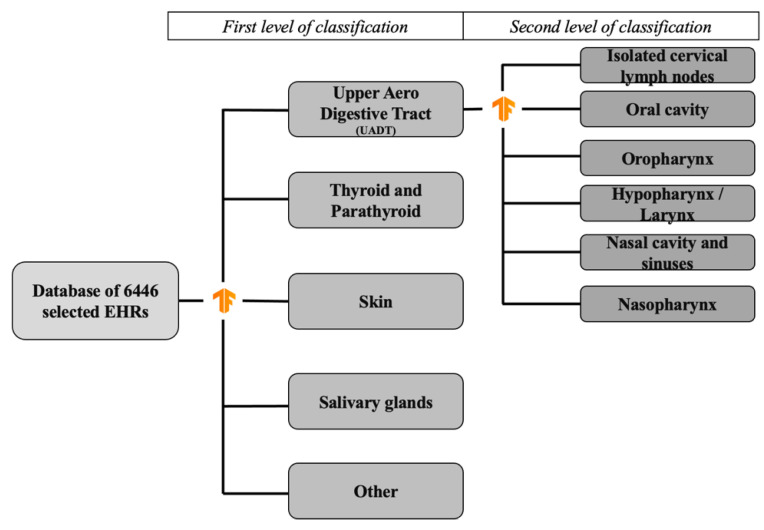
Flow chart of the two levels of classification.

The model was used in two steps for classification of EHR to get as close as possible to human reasoning (Figure 1). In a first step of classification, the model classified EHR according to five categories: “thyroid and parathyroid pathology”, “salivary gland pathology”, “head and neck skin pathology”, “upper aerodigestive tract (UADT) pathology” and “other”. The UADT group, although it corresponds to a well-established clinical entity, actually includes several anatomical locations. We hence applied a second level of classification in six subgroups was performed: “oral cavity”, “hypopharynx/larynx, “oropharynx”, “nasopharynx”, “isolated cervical lymph nodes”, “nasal cavity and sinuses”.

For each group, we analyzed performance according to the metrics defined below.

$$Accuracy=\frac{\mathrm{TP}+\mathrm{TN}}{\mathrm{TP}+\mathrm{TN}+\mathrm{FP}+\mathrm{FN}}$$

$$Recall=\frac{\mathrm{TP}}{\mathrm{TP}+\mathrm{FN}}$$

$$\mathrm{Precision}=\frac{\mathrm{TP}}{\mathrm{TP}+\mathrm{FP}}$$

$$\mathrm{Specificity}=\frac{\mathrm{TN}}{\mathrm{TN}+\mathrm{FP}}$$

$$F1.\mathrm{score}=\frac{2\times \;\mathrm{Precision}\;\times \;\mathrm{Recall}}{\mathrm{precision}\;+\;\mathrm{recall}}$$

where TP is True Positive (TP: the model correctly predicts the label of interest), TN is the True Negative (TN: the model correctly predicts another class than the label of interest), FP is the False Positive (FP: the model incorrectly predicts the label of interest), FN is the False Negative (FN: the model incorrectly predicts another label than the label of interest). We used Confusion matrices to visualize the distribution of patients in the predicted groups based on the actual group (ground-truth labels in ordinate versus model prediction in abscissa).

Based on retrospective and pseudonymized data collection, this study complies Based on retrospective and pseudonymized data collection, this study complies with the French law corresponding to the Methodology of Reference 004 (MR004) for clinical research. A declaration of the database has been made to the French health data hub (N°F20211007110223) and information was given to patients before the start of the study. The study was conducted in compliance with the Helsinki Declaration.

## 3. Results

### 3.1. EHR Extraction and Manual Classification

107,282 head and neck oncology surgery consultation EHR were extracted. From these reports, 24,434 first visits were identified and 6446 (26%) were randomly selected and manually classified by an expert (Head and Neck surgeon) in the ten clinically relevant groups as reported in Table 1.

### 3.2. First Classification Level

We first applied the model on EHR of the first consultation of 6446 patients. The algorithm trained on 5157 EHR (80%) and then tested on 1289 patients (20%).

**Figure 2:**
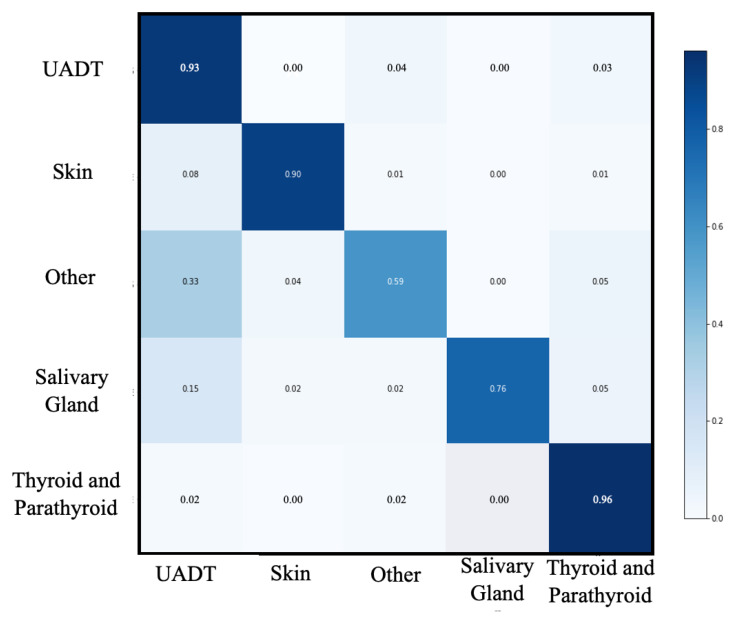
Confusion matrix of first level classification. UADT: upper aerodigestive tract.

Performances of this first level of classification are shown in Table 2 and Figure 2. Accuracy, recall, precision, specificity and F1-score of this step were 0.95, 0.83, 0.85, 0.97 and 0.84, respectively, for the macro-average. The best performance was observed in the “Thyroid and parathyroid” group (accuracy, recall, precision, specificity and F1-score were 0.96, 0.96, 0.95, 0.97, 0.96, respectively). The worst performance was observed in the “other” group (accuracy, recall, precision, specificity and F1-score were 0.93, 0.58, 0.70, 0.97, 0.63, respectively).

### 3.3. Second Classification Level

We applied the same algorithm on the “UADT” group of 2175 patients. The algorithm trained on 1740 EHR (80%) and then tested on 435 patients (20%).

**Figure 3:**
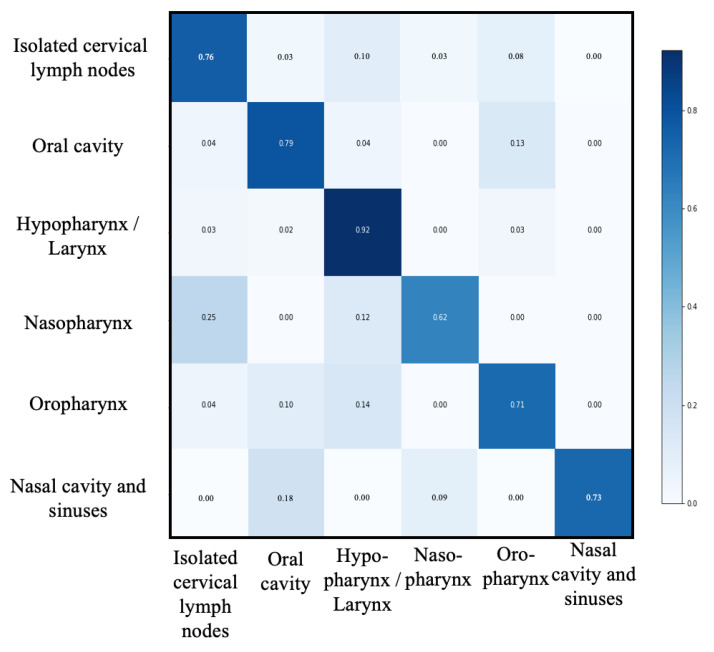
Confusion matrix of second level classification. UADT: upper aerodigestive tract.

Performances of this second level of classification are shown in Table 3 and Figure 3. Accuracy, recall, precision, specificity and F1-score of this step were 0.93, 0.76, 0.83, 0.96 and 0.79, respectively for the macro-average. The best performance was observed in the “Hypopharynx and larynx” group (accuracy, recall, precision, specificity and F1-score were 0.91, 0.91, 0.82, 0.92, 0.86, respectively). The worst performance was observed in the “nasopharynx” group (accuracy, recall, precision, specificity and F1-score were 0.99, 0.63, 0.83, 1, 0.71, respectively).

### 3.4. Extension of the Process

This classification process was then applied to the rest of the cohort of 17 988 patients and the classification results were as follow:

- “Thyroid and parathyroid pathology”: 6798 patients (37.79%)
- “Salivary gland pathology”: 525 patients (2.91%)
- “Head and neck skin pathology”: 2448 patients (13.60%)
- “other”: 1230 patients (6.84%)
- “UADT”: 6987 patients (38.84%)
○ “Oral cavity”: 2083 patients (29.81%)
○ “hypopharynx/larynx”: 2311 patients (33.05%)
○ “oropharynx”: 1379 patients (19.73%)
○ “nasopharynx”: 48 patients (0.69%)
○ “Isolated cervical lymph nodes”: 1062 patients (15.19%)
○ “Nasal cavity and sinuses”: 104 patients (1.48%)

## 4. Discussion

Many different pathologies are involved in the same medical department, so the constitution of patient cohorts with clinical consistency is of major interest. Indeed, the patient cohorts identified are useful for the inclusion of prospective therapeutic trials, for retrospective and real-life studies, and for building local data hubs for clinical or medico-economic evaluation [4,6].

A similar approach with an algorithm based on TensorFlow, an open AI resource, was recently described by Hsu et al. [14]. They applied a convolution neural network model to predict the International Statistical Classification of Disease and Related Health Problems (ICD-9) on subjective component of the progress note of 168,000 medical records of a single center. Previous studies have demonstrated that ICD classification have several limitations and is not sufficient for cohort constitution [15,16,17]. Indeed, for many medical conditions, less than 10% of the affected individuals’ EHR contain the respective International Classification of Diseases (ICD) diagnosis codes [18,19]. ICD can also be miscoded at the time of data analysis or done after data extraction. In fact, diagnostic coding accuracy also varies by setting, provider type, and whether the code was assigned by a billing specialist [20,21,22,23]. Miscoding would lead to measurement error and missing data would contribute to selection bias and neutralize the statistical power available by mining the data contained in HER [12,24,25,26,27].

Therefore, we based our classification on clinically relevant criteria to constitute more homogeneous cohorts according to phenotype. Our classification was based on surgeons’ notes, as physicians’ notes are valuable sources of patient information [28,29].

In this study, we only applied this deep learning approach on first outpatient records to better identify the treated pathology. In addition, we obtained better results than Hsu et al. [14] whose best performance yielded an accuracy of 0.580, a recall of 0.580, and a precision of 0.582 vs. 0.95, 0.83 and 0.85, respectively for macro-average and 0.96, 0.96 and 0.95 for the best group (“Thyroid and parathyroid pathology”) of the first level of classification in our study. The performance of our model was particularly high for the most represented pathology (thyroid pathology) in the first level of classification, explained by a larger training set. Moreover, in the second level of classification, the model achieved acceptable performance in a group where the pathologies involved are anatomically very similar. This classification is however very useful for the data analysis of these patients because even if they can be grouped within the same entity, each of these cancers has a different diagnostic, prognostic and therapeutic management. In addition, we compared the prediction with manually reviewed and labelled data by medical experts, which is considered as the ‘ground truth’ by most of the studies reporting performance on data [13].

Our study provides additional validation in a language other than English [14]. We first applied the process to determine five major and clinically relevant categories. We then used output classification of the UADT group as an input for a second TensorFlow process to determine subcategories in this group. The lower number of output categories gave better performances than a one-step classification.

The data mining approach of Ferrao et al. [9] concluded that logistic regression models had higher performances, while decision trees had a higher precision (lower rate of false positive) and support vector machines a higher recall (lower rate of false negative). Regardless of technique, published performances were poorer for predicting ICD-9 codes than our process to predict the relevant pathology.

Venkataraman et al. [10] approach, based on LSTM RNNs found that the “neoplasia” category gave the best performances among the different categories, explaining our good results as our patients were all in this category. Second, their LSTM RNNs approach had the best performances, with the highest F1-score obtained of 0.91. As a reminder, the F1 score is a score used to evaluate categorization tools in artificial intelligence that takes into account recall and precision. The best result of this study was in line with ours, even if our approach did not include a metamap processing, a clinical natural language processing tool. The authors compared their model to another AI approach, although the gold standard remains a comparison with human-controlled data [11].

For Tam et al. process, it was initially constructed to identify patients with acute coronary syndrome (ACS) in patients in a large general hospital. This model had a high sensitivity but contrary to our model, did not have a second manual or computerized control to confirm the ACS. Manual classification of EHR by medical experts for the learning and testing sets was a strength of our study. In addition, the Tam et al. [12] approach was useful for identifying the phenotype of patients in a primary care medical center. However, this model does not seem suitable for predicting a classification of all patients in a department of a second or tertiary medical center. Our model effectively classified patients with similar phenotypes among patients in the head and neck surgery department.

Hsu et al. [14] had to perform a preprocesing step to standardize Chinese and English text, modify punctuation and perform segmentation. Chiaramello et al. [30] had to perform an English translation from Italian clinical notes with Google Traduction before using the MetaMap steps (tokenization, parsing, variant generation, candidate retrieval, candidate evaluation, and mapping construction). Our approach had the advantage of not requiring a preprocessing step that can be time consuming and lead to errors. EHR were directly and manually classified by medical experts based on the specified pathology to form the input database.

The constitution of patient cohorts based on phenotype and only on the first medical examination could constitute a limitation of our study. Nevertheless, as a tertiary medical center, the locations of neoplasia are most often determined. Otherwise, patients were classified in the “other” group. This group therefore consisted of patients for whom medical expertise was required (patients without tumors) or with an undetermined neoplasia location. Another limitation of this study is that it predicts only one class of pathology involved. Some patients may present with two or more pathologies at the first visit. This is also a limitation for manual classification because our goal was to build cohorts of patients, and only one pathology was labeled for each patient. Even if this event is rare, another neural network could be used to identify patients with multiple cancers at this stage.

The major strength of our study is the open access and ease of use of the resource, which makes this model widely reproducible. Indeed, many authors use AI without a deep understanding of its mathematical, statistical and programming principles [31]. We therefore used this previously reported methodology to validate it in a different center and language than Hsu et al. [14], which can now be easily reproduced elsewhere, and for several different pathologies. The manual classification of EHR for training and validation sets was also a strength of our model, with a limited working time for this step (two days for a head and neck surgeon). This work constitutes an essential first step in the construction of any health data hub. This tool can thus be the first brick of a real time patient data processing. Other research work is necessary to continue the exploitation of the information contained in medical reports in order to extract and structure them.

## 5. Conclusions

We report here a reliable model based on the TensorFlow algorithm, a convolutional neural network with an open resource access, used to build phenotype-based patient cohorts in the head and neck surgery department of a tertiary cancer medical center, without using ICD codes. We report good performances of this process with macro-average performance on the first and second levels of classification of 0.95, 0.83, 0.85, 0.97, 0.84 and 0.93, 0.76, 0.83, 0.96, 0.79 for accuracy, recall, precision, specificity and F1-score. We have therefore validated this previously described approach with a better performance, in another language and without any preprocessing step. This approach represents an easy-to-use tool based on supervised deep learning. In addition, its main limitation was the need of a large initial dataset, with manually reviewed EHR by a medical expert. This model can now be used to build data hubs, to facilitate inclusion in clinical trials, to perform epidemiological and real-life studies, to assist administrative workers for business accounting, and can be applied to other AI models as an automatic data extraction tool. This process should then be applied to all the pathologies treated in our center in order to build real time patient cohort.

## Figures and Tables

**Table 1:** Distribution of patients according to groups in the initial dataset.

Group	Number of Patients (%)
Thyroid and parathyroid pathology	2509 (38.92)
Salivary gland pathology	283 (4.39)
Head and neck skin pathology	841 (13.04)
Oral cavity	618 (9.58)
Hypopharynx/larynx	659 (10.22)
Oropharynx	431 (6.68)
Nasopharynx	38 (0.58)
Isolated cervical lymph nodes	363 (5.63)
Nasal cavity and sinuses	66 (1.02)
Other	638 (9.89)
*Total*	*6446 (100)*

**Table 2:** Algorithm performance for first classification level.

Groups N (%)	UADT	Thyroid and Parathyroid Pathology	Head and Neck Skin Pathology	Other	Salivary Gland Pathology	Macro-Average
	438 (33.4)	538 (41.0)	174 (13.3)	111 (8.5)	51 (3.9)	1312 (100)
**Accuracy**	0.91	0.96	0.98	0.93	0.98	0.95
**Recall**	0.89	0.96	0.92	0.58	0.78	0.83
**Precision**	0.84	0.95	0.93	0.70	0.84	0.85
**Specificity**	0.92	0.97	0.99	0.97	0.99	0.97
**F1-score**	0.87	0.96	0.93	0.63	0.81	0.84

UADT: upper aerodigestive tract.

**Table 3:** Algorithm performance for second classification level.

Groups N (%)	Isolated Cervical Lymph Nodes	Oral Cavity	Oro-Pharynx	Hypopharynx/Larynx	Nasal Cavity and Sinuses	Naso-Pharynx	Macro-Average
	71 (16.3)	120 (27.6)	88 (22.2)	142 (32.6)	8 (1.8)	6 (1.4)	435 (100)
**Accuracy**	0.93	0.90	0.88	0.91	0.99	0.99	0.93
**Recall**	0.77	0.81	0.70	0.91	0.73	0.63	0.76
**Precision**	0.77	0.84	0.73	0.82	1	0.83	0.83
**Specificity**	0.96	0.94	0.93	0.92	1	1	0.96
**F1-score**	0.77	0.82	0.72	0.86	0.84	0.71	0.79
